# The Readiness For Interprofessional Education (IPE) In The School Setting Among The Internship Students Of Applied Medical Sciences At Taibah University

**DOI:** 10.2147/AMEP.S208870

**Published:** 2019-10-03

**Authors:** Suliman Salih, Moawia Gameraddin, Sameer Kamal, Mohamed Alsadi, Jumaa Tamboul, Kamal Alsultan

**Affiliations:** 1 Faculty of Applied Medical Sciences, Taibah University, Medina, Saudi Arabia; 2 National Cancer Institute, University of Gezira, Wad Madani, Sudan; 3 College of Radiological Sciences and Medical Imaging, Alzaiem Alazhari University, Khartoum, Sudan; 4 Diagnostic Radiology Department, Faculty of Applied Medical Sciences, King Abdulaziz Univesity, Jeddah, Makkah, Saudi Arabia

**Keywords:** Interprofessional education, internship student, readiness

## Abstract

**Objective:**

The study aims to assess the readiness of Interprofessional Education (IPE) in the school setting among the Internship Students of Applied Medical Sciences at Taibah University.

**Methods:**

This study utilized a survey targeting internship students of Applied Medical Sciences; departments Diagnostic Radiologic Technology, Medical Laboratory Technology, and Clinical Nutrition, at Taibah University. We used a modified Readiness for Inter-professional Learning Scale (RIPLS) survey to collect the data of this study. Data were analyzed using SPSS software version 21.

**Results:**

A 100 surveys distributed, 40 were returned. Most of the internship students (88.7%) agreed that IPE could make them more collaborated and can enhance teamwork skill to them. A total of 36.25% of the internship student agreed, and 50.01% disagree about the negative professional identity of the IPE. 86.66% of the internship student agreed, and 50.01% disagree about the positive professional identity of the IPE. They responded that sharing learning with other health care professionals will help them to communicate better with patients and other professionals and to improve practice. 65.84% of the internship student agreed, and 23.75% disagreed about the impact of IPE on their role and responsibility. The difference between the internship student at the end level of the internship and internship student at the beginning level of the internship, towards the positive thinking about other healthcare professionals was 0.015.

**Conclusion:**

The study concluded that there was a readiness of IPE in the school setting among the Internship Students Students of Applied Medical Sciences at Taibah University. The majority of the internship student agreed that IPE could make them more collaborated and can enhance teamwork skill to them. There was a significant difference between the internship student at the end level of the internship and internship student at the beginning level of the internship, towards the positive thinking about other healthcare professionals.

## Introduction

Health systems are complex and facing continual challenges and changing across a variety of contexts and health service levels.^1^ The changing in the health services landscape requires more collaboration and teamwork skills between health care professionals to meet the needs of patients care. Coordination approach between health care professionals expected to give better outcomes with lower cost.^2^

Interprofessional education and collaboration may play a partial role to face these challenges and changes in health care system 0.2 IPE has become one of the recommended issues by the World Health Organization (WHO), for improving health care practice and patient safety.^3^ The World Health Organization has initiated action and framework to encourage its member to adopt and implements this type of education among the students of health sciences.^3^ Interprofessional education and collaborative practice are sharing learning and training among health professions.^4^ Previous studies showed that the coordination and collaboration between healthcare professionals have a positive impact on patient outcomes.^5^,^6^ The awareness and implantation of (IPE) plays a critical role in preparing future healthcare providers for more collaboration and coordination.^5^

College of Applied Medical Sciences at Taibah University consists of three departments of Diagnostic Radiologic Technology, Medical Laboratory Sciences, and Clinical Nutrition. There are no formal courses in the curricula of these departments that gather these students together to support IPE and to foster teamwork skills.

Yet, to our knowledge, there have been no studies that addressed students’ readiness for Interprofessional Education (IPE) in the school setting among the internship students from Saudi Arabia in generally and Taibah University in particular. Identifying the need of the students will recognize the importance of IPE in building teamwork among health professional and guide the decision making to introduce this concept in the curricula.

The purpose of this study is to assess the readiness for Interprofessional Education (IPE) in the school setting among the Internship Students of Applied Medical Sciences at Taibah University.

## Methods

### Study Design

This study used a cross-sectional survey design to Assessment of the need for Interprofessional Education (IPE) in the school setting among the Internship Students of Applied Medical Sciences at Taibah University.

### Procedure

The last survey link was emailed and messaged by (Whatsapp) to the participants using the Google form survey. The body of the message in the email or the WhatsApp served as informed consent and contained a Web link that took the participants to the survey in Google form survey. The study remained open for a total of 30 days. We sent reminder messages to all participants 15 days after the initial request. Informed consent was provided when the participants clicked on the link to access the survey. Responses to the study were collected through a google survey facility.

There were two groups of students included in this study. Group one is included the internship students at the beginning level of the internship rotation (first month of rotation) and the second is included the internship students at the end level of the internship rotation (last month of rotation).

Applied Medical Sciences approved the study at Taibah University Review Board.

### Instrumentation

The survey instrument consisted of a general information section and the modified RIPLS, version 2005 which was developed by McFadyen et al.^7^ The general information section assessed participant information such as age, specialty, and internship level. This scale is one of the most widely used instruments in research relating to IPE. It was designed to assess attitudes and perceptions of students regarding IPE The survey composed of 19 items and uses a 5-point Likert scale, and divided into four subscales. The endpoints of the Likert scale are “strongly disagree (1)” to “strongly agree (5).” The four subscales are (1) Teamwork and Collaboration (items1–9, total possible score45); (2) Negative Professional Identity (items10–12, total possible score15); (3) Positive Professional Identity(items13–16, total possible score20); and (4) Roles and Responsibilities s(items17–19,total possiblescore15).Test-retest reliability for the RIPLS was examined in McFadyen et al and found to be acceptable for both subscales and individual items,^7^ as in the link https://goo.gl/forms/vmTIt63IKDxjiM5Z2.

There were two groups of students included in this study. Group one included 21 students who they are at the beginning level of the internship rotation (first month of rotation). While the second group included 19 students at the end level of the internship rotation (last month of rotation) level of the internship rotation (21 students) Surveys were sent electronically by emails a link to all students for the same two-week period using the Google form survey. The response rate was 40% for all participants. Completion of the study was voluntary. In addition to the 19 items of the RIPLS survey, the researchers asked students about specialty (medical laboratory technology, radiologic technology, and clinical nutrition) and gender. Data were analyzed using SPSS software version 21. Approval for the study was granted by the Colleges of Applied Medical Sciences ethical committee Review Board, at Taibah University.

Data were collected during the second half of 2016 from all applied medical sciences internship students at Taibah University, including internship students of Diagnostic Radiologic Technology, Medical Laboratory Technology, and Clinical Nutrition.

### Data Analyses

Data was entered and analyzed data using SPSS software (version 21.00). Descriptive statistics were used for calculating the frequency of continuous variables. Chai squire test was used to analyze categorical variables.

## Results

A hundred surveys were distributed; among which 40 ones returned, (40% response rate). There were 37.5% from a Medical Laboratory Technology interns, 32.5% from diagnostic radiologic technology interns, and 30% from clinical nutrition interns. There were 21 internship students at the beginning level of the internship rotation (first month of rotation), and there were 19 internship students at the end level of the internship rotation (last month of rotation) level of the internship rotation.

Table 1 summarized the frequency analyses calculation. It revealed that the majority of the internship students (88.7%) agreed that IPE can make them more collaborated and can enhance teamwork skill to them, 20% undecided and 13.7% disagree 0.33% of the internship student disagreed, 17.5% uncertain and 50.01% disagree about the negative professional identity of the IPE, and they thought it was a waste of time and not necessary.

**Table 1:** Evaluation Of Subscales According To Students, Response

Subscales	Response Of Students						
Strongly Agree	Agree	Total Frequency Agree (Strongly Agree And Agree)	Undecided	Disagree	Strongly Disagree	Total Frequency (Disagree And Strongly Disagree)
Teamwork and collaboration	184 (51%)	118 (32.78%)	88.78%	30 (20%)	24 (8.57%)	4 (5%)	13.57%
Negative professional ID	33 (20.62%)	26 (16.25%)	36.25%	21 (17.5%)	53 (33.13%)	27 (16.88%)	50.01%
Positive professional ID	64 (53.33%)	44 (33.33%)	86.66%	10 (8.33%)	1 (2.5)	1 (2.5%)	5%
Roles and responsibility	41 (34.17%)	39 (31.67%)	65.84	22 (13.33%)	13 (16.25%)	6 (7.5%)	23.75%

An 86.66% of the internship students agreed, 17.5% undecided and 50.01% disagree about the positive professional identity of the IPE, and they responded that sharing learning with other health care professionals will help them to communicate better with patients and other professionals and to improve practice. 65.84% of the internship students agreed, 13.33% undecided, and 23.75% disagreed about the impact of IPE on their role and responsibility.

Table 2 summarizes the four subscales in departments of Diagnostic Radiology, Medical Laboratory, and Clinical Nutrition. The relationship between subscales and the disciplines of internship students was statistically insignificant.

**Table 2:** The Relationship Between Subscales And Disciplines Of Students

Subscales	Diagnostic Radiologic Technology N=12	Medical Lab. Technology N=15	Clinical Nutrition N=13	Frequency	p-Value
Teamwork and collaboration	1.94 (0.99)	1.69 (0.93)	1.60 (0.68)	0.888	0.868
Negative professional ID	3.58 (1.24)	3.47 (1.50)	3.69 (1.18)	0.148	0.868
Positive professional ID	1.67 (0.60)	1.68 (0.92)	1.49 (0.57)	0.422	0.66
Roles and responsibility	2.25 (1.08)	2.27 (1)	2.10 (0.84)	1.00	0.48

**Note:** P-values < 0.05% were significant.

The question “Shared learning will help me think positively about other healthcare professionals” is statistically different between the group of students at the end level of internship period and students who were at the beginning of the internship period. (P-value= 0.015). On the other hand, the result showed a significant difference between the two groups in answering the question “learning to work, students/professionals need to respect and trust each other” p-value = 0.033 as shown in Table 3. (There was a significant difference between the internship student at the end level of the internship and internship student at the beginning level of the internship, towards the positive thinking about other healthcare professionals).

**Table 3:** The Attitude Of Internship Students Towards Teamwork And Collaboration

Status		N	Mean	SD	P-value
Shared learning will help me think positively about other healthcare professionals	Student at The beginning level of the internship	21	1.7619	0.94365	0.015
Student at The end level of the internship	19	2.7368	1.44692
Learning to work, students/professionals need to respect and trust each other	Student at The beginning level of the internship	21	1.7619	1.13599	0.033
Student at The end level of the internship	19	2.6316	1.34208

**Note:** P-values < 0.05% were significant.

## Discussion

The IPE is essential in improving collaborative practice, teamwork skills, and improving patient outcomes.^8^ The readiness for Interprofessional Education (IPE) in the school setti.ng among the Internship Students of Applied Medical Sciences at Taibah University, remains unknown. The current research attempted to address the question as to whether the Internship Students of Applied Medical Sciences at Taibah University are ready for Interprofessional Education (IPE) in the local context? To answer this question we used modified RIPLS, version 2005 which was developed by McFadyen et al^7^ However, Other studies have reported the limitation RIPLS’ for students of health professional at different stages of their educational/professional career,^9^,^10^ the modified RIPLS version we used it in one stage (undergraduate), can be useful as a baseline study.

The results of this study found that internship students of Applied Medical Sciences were both ready and willing to participate in IPE. The majority of them (88.78%) thought that learning with other student’s professionals will make them more useful members of a health care team and more collaborated. This finding is in agreement with many studies reported that interprofessional education could build confidence among health care professionals and foster collaboration.^4^,^5^ Cooperation and teamwork are essential to improve health service and care of patients worldwide. However, awareness IPE during health professional’s education will shape their future practice from the early stages of training.^11^,^12^ Implantation of IPE in the health professional students, need to build these concepts in the curriculum health care schools setting to increase the awareness of the faculty and clinical instructors involved in teaching.^12^ Many studies reported that a lack of collaboration between health professional was related to the misunderstanding health care professional with the role and scope of practice with each other.^13^,^14^

The study showed that the question “Shared learning will help me think positively about other healthcare professionals” was statistically different between the group of students at the end of internship period (last month) than those who at the beginning level(first month) of the internship period, (p-value= 0.015). The study revealed that the question “for small group learning to work, students/professionals need to respect and trust each other” was statistically different between the group of students at the end of internship period than those who at the beginning level of internship period p-value = 0.033 as shown in Table 3. The reason that this group had experience in hospital practice, and they faced many situations and barriers regarding other professional, and the needs to understand others. It thought that health care professionals possess an insufficient understanding of one another’s contributions. Thus, traditional role perceptions are sustained.^15^ Similarly, Curran et al, and Rebecca Olson and Andrea found that “significant differences in the attitudes of health sciences students from different professions continue to persist”.^16^,^17^

The relationship between subscales and discipline of students is insignificant. We can explain this finding, that the majority of the participants had no direct contact with the colleagues and the health care system, such as clinical nutrition and medical laboratory intern students. Mark et al who reported that students who had no exposure to the health care system, either as a patient or as a family member of a patient, had a lower score on the Negative Professional Identity subscale.^18^,^19^

We found that only 36.25% of the students have a negative professional towards learning with a health care professional, 50% disagree with negative professional, and they thought that learning with other health care students is a useful Table 1. On the contrary, it has been reported in the literature that observation of staff behaviors can impact negatively on the students’ discipline, attitudes, values, and practices.^19^,^20^ In the same context another study by Makino et al.^21^ assessed the relationship between exposure to clinical practice and attitudes towards interprofessional healthcare teams using the modified Attitudes towards Healthcare Teams Scale (ATHCTS). They found that alumni had significantly lower overall mean scores than undergraduate students, inferring that exposure to clinical practice may distract students away from the positive attitudes towards the efficacy of healthcare teams.^21^

## Study Limitations

The limitations of this study are; the low response rate for the survey, the small sample size, and the data were taken from one institution

## Conclusion

The study concluded that there is a need for Interprofessional Education (IPE) in the school setting among the Internship Students of Applied Medical Sciences at Taibah University. We found that learning with other health professionals will make the health care professional more collaborated with other and foster teamwork skill to them. There was a significantly different between the internship student at the end level of the internship and internship student at the beginning level, towards the positive thinking about other healthcare professionals.

### Recommendations

The authors recommend a further study with large sample size and high response rate to confirm these findings and generalize the conclusion. The study recommended introducing IPE in curricula of the College of Applied Medical Sciences at Taibah University.
